# Exosomes derived from programmed cell death: mechanism and biological significance

**DOI:** 10.1186/s12964-024-01521-0

**Published:** 2024-03-01

**Authors:** Min Xiong, Zhen Chen, Jiaqi Tian, Yanjie Peng, Dandan Song, Lin Zhang, Yulan Jin

**Affiliations:** 1 School of Public Health, North China University of Science and Technology, Tangshan, 063000 China; 2 Clinical Medical Research Center for Women and Children Diseases, Key Laboratory of Birth Regulation and Control Technology of National Health Commission of China, Shandong Provincial Maternal and Child Health Care Hospital Affiliated to Qingdao University, Jinan, 250001 China; 3 Key Laboratory of Birth Defect Prevention and Genetic Medicine of Shandong Health Commission, Jinan, 250001 China; 4 Hebei Key Laboratory of Coal Health and Safety, Tangshan, 063000 China; 5 School of Public Health, Weifang Medical University, Weifang, 261000 China

**Keywords:** Exosome, Programmed cell death, Biogenesis, Regulatory mechanisms

## Abstract

Exosomes are nanoscale extracellular vesicles present in bodily fluids that mediate intercellular communication by transferring bioactive molecules, thereby regulating a range of physiological and pathological processes. Exosomes can be secreted from nearly all cell types, and the biological function of exosomes is heterogeneous and depends on the donor cell type and state. Recent research has revealed that the levels of exosomes released from the endosomal system increase in cells undergoing programmed cell death. These exosomes play crucial roles in diseases, such as inflammation, tumors, and autoimmune diseases. However, there is currently a lack of systematic research on the differences in the biogenesis, secretion mechanisms, and composition of exosomes under different programmed cell death modalities. This review underscores the potential of exosomes as vital mediators of programmed cell death processes, highlighting the interconnection between exosome biosynthesis and the regulatory mechanisms governing cell death processes. Furthermore, we accentuate the prospect of leveraging exosomes for the development of innovative biomarkers and therapeutic strategies across various diseases.

## Introduction

Exosomes are phospholipid vesicles ranging from 30 to 150 nanometers in length that are secreted by cells to facilitate intercellular communication through biomolecular transfer [1]. Exosomes were first observed in vitro in 1983 from cultured sheep reticulocytes and were subsequently formally named ‘exosomes’ by Johnstone in 1987. Exosomes were initially considered cellular waste products but are now recognized as pivotal in signaling and ubiquitously found in bodily fluids. Exosome generation, secretion, and molecular composition intricately depend on factors such as cell type, growth status, and receptor stimulation. For instance, kinetic and compositional differences exist between exosomes from white blood cells and red blood cells. Conditions such as oxidative stress or inflammation notably alter leukocyte exosome content [2]. Bioactive compounds, such as proteins and RNAs, encapsulated in exosomes released from cells undergoing death differ from those released by normal viable cells [3].

Cell death, including regulated processes such as apoptosis, necroptosis, autophagy, pyroptosis, and unregulated necrosis triggered by external factors, is crucial for cellular, tissue, and organismal homeostasis [4]. Despite varying initiation mechanisms, each programmed cell death form significantly influences exosome release, bioactive molecule encapsulation, and intercellular communication [3]. Exosomes from different cell death pathways play distinct roles; for example, those from apoptotic cells may activate immune responses [5], while those from necrotic or pyroptotic cells can induce immune cell death and amplify inflammation [6].

This review aims to consolidate the current understanding of the mechanisms, compositions, and functions of exosomes released during programmed cell death. Investigating these aspects, particularly in the context of regulated cell death, opens new avenues for disease diagnosis and targeted therapeutics. Overall, this review emphasizes the critical role of exosomes as regulators of communication between dying and healthy cells, highlighting their importance in cellular biology.

### Biological functions of exosomes

#### Intercellular communication and signal transduction

**Fig. 1:**
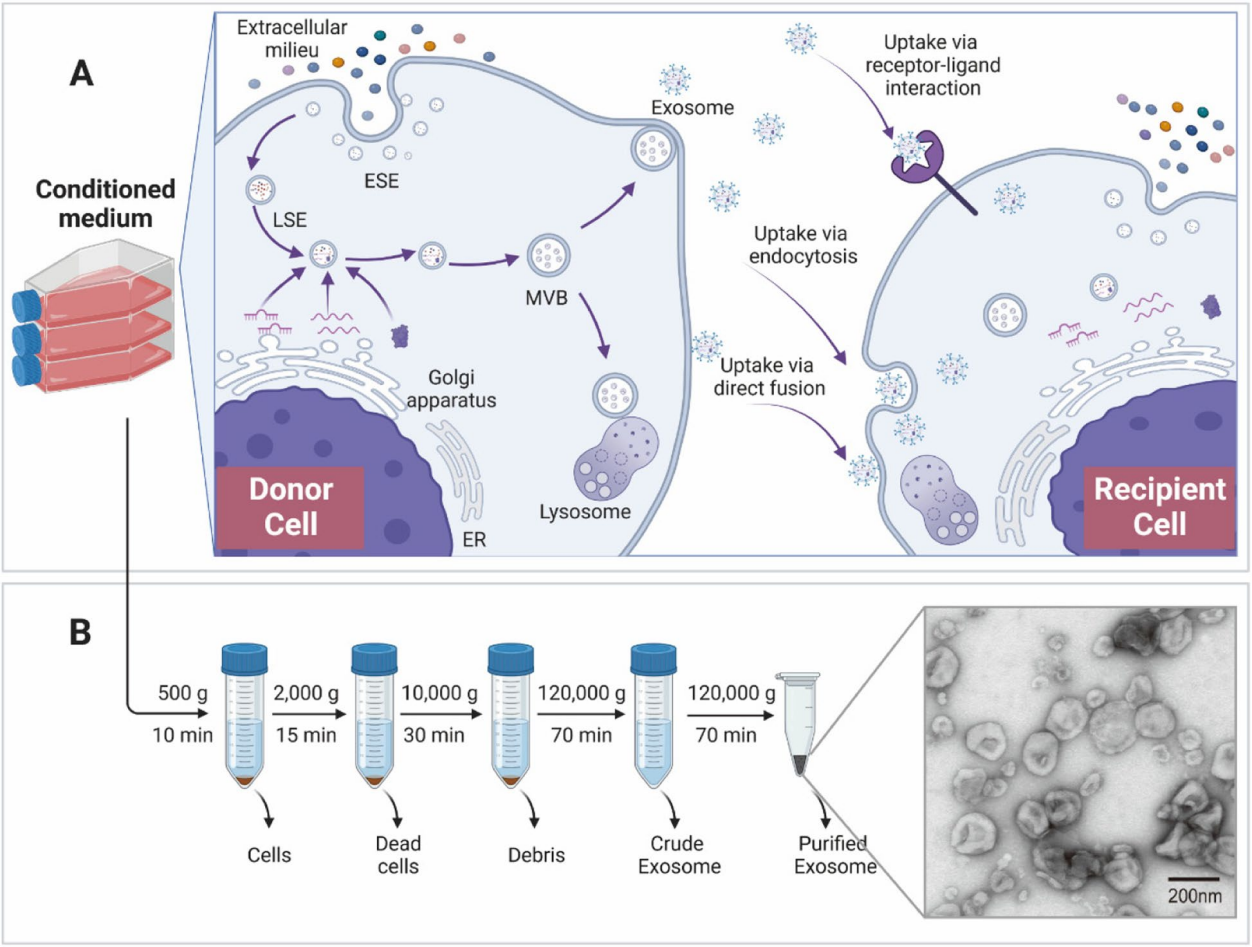
Exosome biogenesis, secretion, uptake, and isolation. **A** Exosome biogenesis occurs within the endosomal system and involves crucial organelles such as mitochondria, the endoplasmic reticulum, and the Golgi apparatus. It commences with plasma membrane invagination, forming early endosomes (ESEs), which subsequently fuse into late endosomes (LSEs). At this juncture, LSEs participate in cargo sorting near the Golgi apparatus, resulting in the generation of multivesicular bodies (MVBs). These MVBs can undergo lysosomal degradation, intracellular recycling, or secretion as exosomes. Once secreted, recipient cells internalize exosomes through mechanisms such as receptor-mediated endocytosis, phagocytosis, or direct fusion with the plasma membrane. **B** Differential centrifugation is a standard technique for isolating exosomes from conditioned cell culture media. Subsequent purification of the exosomes was achieved via ultracentrifugation at 120,000 × g. The resulting purified exosome suspension was subsequently applied to copper grids for sedimentation, followed by uranyl acetate double staining. The exosomes were subsequently visualized through transmission electron microscopy. The purified exosomes displayed a cup-shaped morphology, ranging in size from 30 to 150 nm, as observed in microscopy images (transmission electron microscopy images derived from exosomes secreted by J774A.1 macrophages extracted by our research group)

Exosomes, which serve as vital intercellular messengers, facilitate the conveyance of bioactive molecules to nearby target cells via paracrine secretion or distally through the circulatory system. They convey contents to recipient cells through mechanisms such as membrane fusion, receptor-mediated endocytosis, or phagocytosis. Owing to their size limitations, noncoding RNAs, including miRNAs, circRNAs, and lncRNAs, predominate within exosomes (Fig. 1). These exosomes play a significant role in regulating gene transcription in recipient cells by transporting noncoding RNAs. For instance, Jumo et al. reported that exosomes from nicotine-stimulated macrophages containing miR-21-3p promote the proliferation and migration of vascular smooth muscle cells (VSMCs) via the PTEN pathway, thereby accelerating atherosclerosis [7]. Similarly, Yin Hu et al. demonstrated that exosomes containing miR-21-3p enhance fibroblast activation and angiogenesis in skin wound healing [8]. Additionally, a considerable number of circRNAs are present in exosomes; for example, tumor-derived exosomes containing circPACRGL can augment colorectal cancer cell growth, migration, and invasion through the miR-142-3p/miR-506-3p-TGF-β axis [9].

In summary, exosomes, as minuscule cell-derived vesicles, play a crucial role in transmitting intracellular information to adjacent environments or other cells. The primary roles of these proteins in cell biology and physiology include intercellular communication and signal transduction.

#### Immune regulation

Exosomes serve as versatile biological regulators of immune responses by employing diverse mechanisms. First, most immune cells (e.g., dendritic cells, B lymphocytes, T lymphocytes, neutrophils, and mast cells) serve as donor cells in immunoregulation by releasing exosomes carrying signaling molecules. For instance, dendritic and B-cell-derived exosomes function as antigen-presenting platforms, priming and activating T cells [10]. Notably, exosomes exhibit functional heterogeneity; those secreted by identical immune cells may exert varied impacts on the immune microenvironment, influenced by compositional differences induced by distinct stimuli. For instance, Yili Wang et al. reported that exosomes isolated from M2 macrophages, which contain mRNAs related to the PI3K/AKT pathway, could reprogram M1 macrophages to the M2 phenotype, functioning as immunomodulators and expediting diabetic fracture healing [11]. Conversely, Fuquan Zhang et al. reported that M2 macrophage-derived exosomes, which are rich in the long noncoding RNA AGAP2-AS1, enhance the efficacy of radiotherapy by inhibiting miR-296 and augmenting NOTCH2 signaling [12]. Hence, the impact of exosomes on immune regulation relies primarily on their bioactive substance content.

Moreover, exosomes facilitate downstream communication among immune cells and modulate the immune microenvironment. Specific exosomes encapsulate immunomodulatory substances such as major histocompatibility complex (MHC), costimulatory molecules, and other immunomodulators that interact with mammalian immune cell receptors, influencing their activities and functionalities [13]. Additionally, exosomes contain regulatory RNAs, including miRNAs and lncRNAs, which bind to target genes and mediate signaling pathways affecting immune cell operation and overall immune system dynamics [10]. For instance, adipose stem cell-derived exosomes can alleviate LPS-induced inflammation and provide protection against sepsis by modulating Nrf2/HO-1 expression [14]. Similarly, mesenchymal stem cell-derived exosomes can mitigate oxidative skin damage by adaptively regulating the NRF2 defense system [15]. Exosomes can also modulate innate immune responses and be engineered into therapeutic agents and vaccines for cancer, suggesting promising applications in the diagnosis, treatment, and prognosis of cancer. For instance, exosomes enriched in tumor antigens such as MHC I may serve as acellular vaccines to prime antitumor immune responses for cancer immunotherapy [16]. Moreover, exosomes harboring heat shock proteins (HSPs) and programmed death ligand 1 (PD-L1) can inhibit immune cell function and deplete the immune repertoire to facilitate immune evasion. Blocking the generation of such immunosuppressive exosomes has also emerged as a novel immunotherapeutic strategy [17].

In summary, exosomes are pivotal for immune system regulation through processes such as encapsulation, secretion, and uptake, offering novel insights and therapeutic avenues for treating immune-related disorders.

#### Inflammation regulation

Exosomes play a pivotal role in orchestrating inflammatory responses by transmitting inflammatory factors and activating downstream target cells through antigen presentation. For instance, immune cells release exosomes, which contain anti-inflammatory agents such as interleukin-10 (IL-10) and transforming growth factor-β (TGF-β), during inflammatory reactions, effectively curbing excessive inflammation [18]. Additionally, microRNAs (miRNAs) within exosomes downregulate the expression of inflammatory genes, including NF-κB and TNF-α, contributing to inflammation. Notably, bone marrow-derived macrophage exosomes carrying miR-99a/146b/378a target these pathways, reducing inflammation and inducing an anti-inflammatory M2 phenotype in recipient macrophages, thereby modulating both the intensity and duration of inflammatory responses [19].

Exosomes also exhibit dual functions in regulating inflammation. Inflammasome activation influences exosome release; conversely, exosomes can modulate inflammasome activity to either promote or suppress inflammation [20, 21]. For instance, upon exposure to zinc oxide nanoparticles (ZnONPs) and UVB radiation, pyroptotic cells release exosomes containing the NLRP3 inflammasome, extending damage to adjacent cells [22]. Overexpression of thioredoxin-interacting protein (TXNIP) activates TLR4/NF-κB/NLRP3 signaling, inducing pyroptosis in rat cardiomyocytes. However, exosomes from M2 macrophages, which carry microRNA-148a, downregulate TXNIP and impede NLRP3 inflammasome activation, thereby alleviating myocardial ischemia/reperfusion injury [23].

In summary, exosomes play a crucial role in orchestrating inflammatory responses by transporting inflammation-related factors, triggering downstream inflammatory signaling, and influencing inflammasome activity. The inflammatory impact of exosomes varies with the mode of cell death they originate from; exosomes from dying cells often exhibit more pronounced proinflammatory properties than those from viable cells. This underscores the importance of gaining a deeper understanding of how different cell death modalities and exosome release interplay in inflammation regulation, offering potential avenues for developing therapeutic strategies.

#### Tumor microenvironment (TME) mediation

Cancer cell-derived exosomes play a crucial role in mediating communication with neighboring cells, establishing a feedback loop that modulates the tumor microenvironment (TME) and fosters tumor growth, invasion, and metastasis [24]. Research by Selma Maacha et al. demonstrated that exosomes can act as carriers of carcinogens, and inhibiting their release effectively reduces carcinogen transfer [25]. Specifically, exosomes from tumors enriched with epithelial–mesenchymal transition (EMT)-related biomolecules such as TGF-β, hypoxia-inducible factor (HIF) 1α, β-catenin, and miR-23a induce EMT in cancer cells, leading to stromal remodeling and degradation [26]. This process is pivotal for tumor cell invasion, migration, and distant metastasis. Additionally, exosomes therapeutically influence the TME by impacting pathways related to intercellular communication, the immune response, matrix remodeling, and drug resistance. For example, Zhang Wei et al. reported that tumor cell-derived exosomes containing programmed cell death ligand 1 (PD-L1) can bind to programmed cell death protein 1 (PD-1) on CD8 + T cells, inhibiting T-cell activity, facilitating immune escape, and promoting tumor immune tolerance [27]. Exosomes derived from tumor cells contain various microRNAs, long noncoding RNAs, and circular RNAs that can promote tumor progression by inducing angiogenesis. However, some noncoding RNAs may conversely inhibit tumorigenesis. Targeting proangiogenic noncoding RNAs in exosomes could be a promising therapeutic strategy for cancer treatment [28]. Mesenchymal stem cell-derived exosomes reduce cancer cell drug resistance and enhance treatment efficacy [29]. Exosomes derived from mesenchymal stem cells (MSCs) can serve as excellent drug delivery vehicles for targeted cancer therapy. For instance, drugs can be loaded into purified MSC-derived exosomes (MSC-EVs) via direct insertion or coincubation methods. Engineered MSC-EVs can increase the affinity and uptake of target cancer cells. Ultimately, these optimized MSC-EVs may act as ideal drug carriers for the treatment of cancers and other diseases [30].

The development and application of therapeutic exosomes in precision medicine present promising prospects for tumor therapy, introducing innovative strategies for cancer treatment. Recent studies suggest that encapsulating exosomes with antitumor drugs or genes is an innovative approach for targeted cancer therapy [31]. Modifying the bioactive components of exosomes through genetic or pharmacological methods can selectively promote or inhibit tumor growth for therapeutic purposes. For example, exosomes loaded with miR-126 can initiate protective autophagy and programmed cell death in cancer cells, producing anticancer effects [32]. The role of exosomes in tumor progression varies with different programmed cell death pathways; for example, exosomes rich in apoptosis-inducing factor (AIF) may facilitate tumor cell apoptosis [32, 33], whereas caspase-mediated exosomes could enhance cancer cell proliferation and invasion [34, 35].

Therefore, understanding the mechanisms of exosome release in specific programmed cell death pathways and their biological functions in cancer is crucial for advancing our understanding of programmed cell death and progression in cancer diagnosis and targeted therapy.

### Classification and mechanisms of programmed cell death modes

#### Apoptosis

Apoptosis, the earliest recognized form of programmed cell death, plays a crucial role in maintaining cellular homeostasis. It encompasses distinctive morphological alterations, including membrane reshaping, protuberances, cell shrinkage, and nuclear chromatin condensation. Apoptotic processes involve DNA fragmentation, mitochondrial dysfunction with increased permeability, and the release of cytochrome c (Cyt c) into the cytoplasm, culminating in the formation of apoptotic bodies [36]. These apoptotic bodies, which contain intracellular proteins and nucleic acids, signal neighboring cells for elimination. Phosphatidylserine exposure on apoptotic bodies allows phagocytes, such as macrophages, to engulf them. The removal of apoptotic bodies by phagocytes prevents excessive inflammation and mediates immune responses. Additionally, apoptotic cells release exosomes, a specific subclass of extracellular vesicles with distinct biogenic origins and functions compared to apoptotic bodies.

The initiation of apoptosis entails caspase enzyme activation and cascade mediation. Procaspase enzymes undergo proteolytic cleavage, activating initiator caspases (caspase-2, -8, -9, and − 10) and executioner caspases (caspase-3, -6, and − 7), leading to irreversible cell disassembly and death [37, 38]. Apoptosis can be triggered through three primary pathways: extrinsic (death ligand‒receptor binding), intrinsic (mitochondria-dependent), and endoplasmic reticulum (ER) stress-related [39].

In the extrinsic pathway, which is initiated by death ligands (FasL, TNF-α, TRAIL) binding to death receptors (FASRs, TNFR1), signaling cascades are activated, ultimately leading to apoptosis [40]. Death receptors, which have extracellular cysteine domains and intracellular death domains, form the death-inducing signaling complex (DISC), which activates caspases-8 and − 10. These caspases then activate caspase-3 and − 7, resulting in the degradation of essential cellular proteins and the induction of apoptosis [41]. Exosomes can also induce apoptosis in recipient cells through the extrinsic pathway by transporting death receptor-related molecules or regulators [42, 43].

The intrinsic mitochondrial pathway is regulated by the balance between proapoptotic and antiapoptotic proteins [44]. Apoptotic stimuli suppress antiapoptotic proteins belonging to the BCL2 family (e.g., BCL-2, BCL-xL, and MCL-1) while activating proapoptotic BH3 proteins (e.g., BID, BIM, and PUMA). This process induces conformational changes and the assembly of the BAX and BAK proteins. These proteins assemble into pore structures in the outer mitochondrial membrane, causing alterations in the mitochondrial membrane potential, increased permeability, and induction of mitochondrial outer membrane permeabilization (MOMP) [38]. MOMP leads to the release of proapoptotic mitochondrial contents, such as cytochrome c (Cyt c), into the cytoplasm, thereby facilitating apoptotic processes. Cyt c binds to Apaf-1, resulting in the formation of the apoptosome and the activation of caspase-9. Subsequently, caspase-9 triggers the executioner caspases (caspase-3, -6, and − 7) in the apoptotic cascade [44]. Activated caspase-3 not only governs apoptosis but also facilitates the release of extracellular vesicle-like exosomes (ApoExos), influencing recipient cells [45]. These exosomes may influence mitochondrial membrane proteins via miRNA release, affecting intrinsic pathway activity and mitochondrial membrane permeability, or they may directly transmit molecules such as Cyt c to modulate apoptosis [46, 47].

The endoplasmic reticulum (ER) serves as the primary intracellular organelle for calcium storage, protein synthesis, and protein modification. Elevated ER stress, resulting from calcium imbalance or the accumulation of misfolded proteins, triggers apoptosis via the ER stress-induced cell death pathway [48]. Furthermore, ER stress facilitates the release of exosomes, which subsequently modulate the activity of the ER stress pathway by influencing molecules such as PERK and ATF6 [49, 50].

#### Necroptosis

Necroptosis, triggered by severe stimuli such as hypoxia, viral infection, chemical toxins, or radiation damage, represents an inflammatory form of cell death. It is characterized by necrosis, such as cytoplasmic leakage, organelle swelling, plasma membrane rupture, cell lysis, and disintegration. This process results in the release of danger-associated molecular patterns (DAMPs), including cytochrome c, disrupting the intracellular environment and triggering immune activation, ultimately leading to tissue damage [51–53]. In contrast to conventional necrosis, necroptosis is governed by distinct regulatory mechanisms.

The necroptotic pathway entails sequential phosphorylation of crucial proteins, specifically receptor-interacting serine/threonine-protein kinase 1 (RIPK1), receptor-interacting serine/threonine-protein kinase 3 (RIPK3), and mixed lineage kinase domain-like pseudokinase (MLKL) [54]. RIPK1, serving as a pivotal initiator, associates with RIPK3 to form and phosphorylate the necrosome. Subsequently, activated RIPK3 phosphorylates MLKL, resulting in its oligomerization and the formation of pores in the plasma membrane. This cascade leads to calcium influx, membrane rupture, and necroptosis [55]. In certain scenarios, necroptosis can occur independently of RIPK1. Z-DNA binding protein 1 (ZBP1), a crucial sensor of necroptosis, can directly bind RIPK3 and MLKL through its RHIM domain, activating RIPK1-independent necroptosis [56]. Therefore, RIPK3/MLKL are central to the execution of necroptosis, independent of caspases [51]. Moreover, necroptosis can be facilitated by the PANoptosome complex, in which ZBP1 and the RIPK3 complex recruit caspase-8, activating the ZBP1-NLRP3 inflammasome and orchestrating the PANoptotic process. This complex is involved in both pyroptosis and apoptosis [57]. In addition to its role in mediating cell death, RIPK3/MLKL also impacts exosome release, as these components are encapsulated in exosomes and transferred to recipient cells [58].

#### Autophagy

Autophagy, a cellular mechanism for self-digestion and degradation, represents a form of programmed cell death. Various stressors, including starvation, radiation, hypoxia, bacterial invasion, and growth factor deficiency, can induce autophagy, which is characterized by cytoplasmic vacuolization and the formation of autophagosomes. After the fusion of autophagic vacuoles and phagosomes, autophagosomes engulf cellular components for subsequent degradation. Microtubules play a crucial role in transporting autophagosomes to lysosomes for degradation. Unlike apoptosis, autophagy is governed by autophagy-related (ATG) genes. These genes selectively target damaged organelles and protein aggregates for lysosomal digestion, preventing cell damage [59].

The initiation of autophagy is catalyzed by the activation of the ULK1 complex, which is composed of ULK1, FIP200, and ATG proteins (ATG12 and ATG101). This complex phosphorylates PI3KC3, resulting in the formation of the ULK1-PI3KC3 complex, subsequently activating PI3P. During this process, additional ATGs, including ATG5, ATG12, ATG16L1, and LC3, are recruited. Simultaneously, LC3-I is phosphorylated, generating lipidated LC3-II on autophagosomal membranes—an indispensable marker of autophagy initiation [60].

Autophagy encompasses macroautophagy, microautophagy, and chaperone-mediated autophagy (CMA) [61]. Macroautophagy entails the sequestration of large cytoplasmic portions in autophagosomes. These structures then fuse with lysosomes to create autolysosomes for subsequent degradation and recycling. Microautophagy directly engulfs cytosolic components through invagination of the lysosomal membrane. CMA depends on Hsc70 to recognize and translocate specific substrate proteins into lysosomes for degradation [62].

In summary, autophagy is an intracellular catabolic process in which autophagosomes encapsulate and degrade harmful or redundant substances into recyclable biomolecules. Autophagy plays a vital role in waste removal, providing energy and nutrients, and regulating cellular differentiation, development, and immunity. Exosomes derived from autophagy, distinct from those released by autophagosomes, transport autophagic signals and bioactive cargo in an ATG-dependent manner [63].

#### Pyroptosis

Pyroptosis, a form of programmed inflammatory cell death, is characterized by both apoptosis and necrosis. It initiates chromatin condensation, followed by plasma membrane rupture, cell swelling, and lysis [64].

Pyroptosis can be divided into classical and nonclassical pathways. The classical pathway, which is dependent on caspase-1, is triggered by pathogen- or danger-associated molecular patterns (PAMPs/DAMPs). This pathway results in NLRP3 oligomerization, recruitment of pro-caspase-1, and the expression of apoptosis-associated speck-like protein (ASC), ultimately leading to the formation of the NLRP3 inflammasome. Activated caspase-1 cleaves pro-IL-1β/IL-18 into mature cytokines and gasdermin D (GSDMD), generating a pore-forming N-terminal fragment. This fragment integrates into the plasma membrane, forming pores that induce cell swelling, lysis, and release of IL-1β/IL-18 [6, 64]. The nonclassical pathway, initiated by LPS, activates human caspase-4/5 or mouse caspase-11, resulting in GSDMD cleavage and subsequent cytokine release [65–67]. Pyroptotic cells release inflammatory DAMPs, including IL-1β, IL-18, HMGB1, ATP, and DNA, intensifying inflammation in neighboring cells [68].

Inflammasome-mediated pyroptosis entails the activation of the NLRP3 inflammasome, leading to cell death and the release of inflammatory exosomes. These exosomes encapsulate molecules that enhance immune responses, inflammation, and phagocytosis [69, 70].

**Fig. 2:**
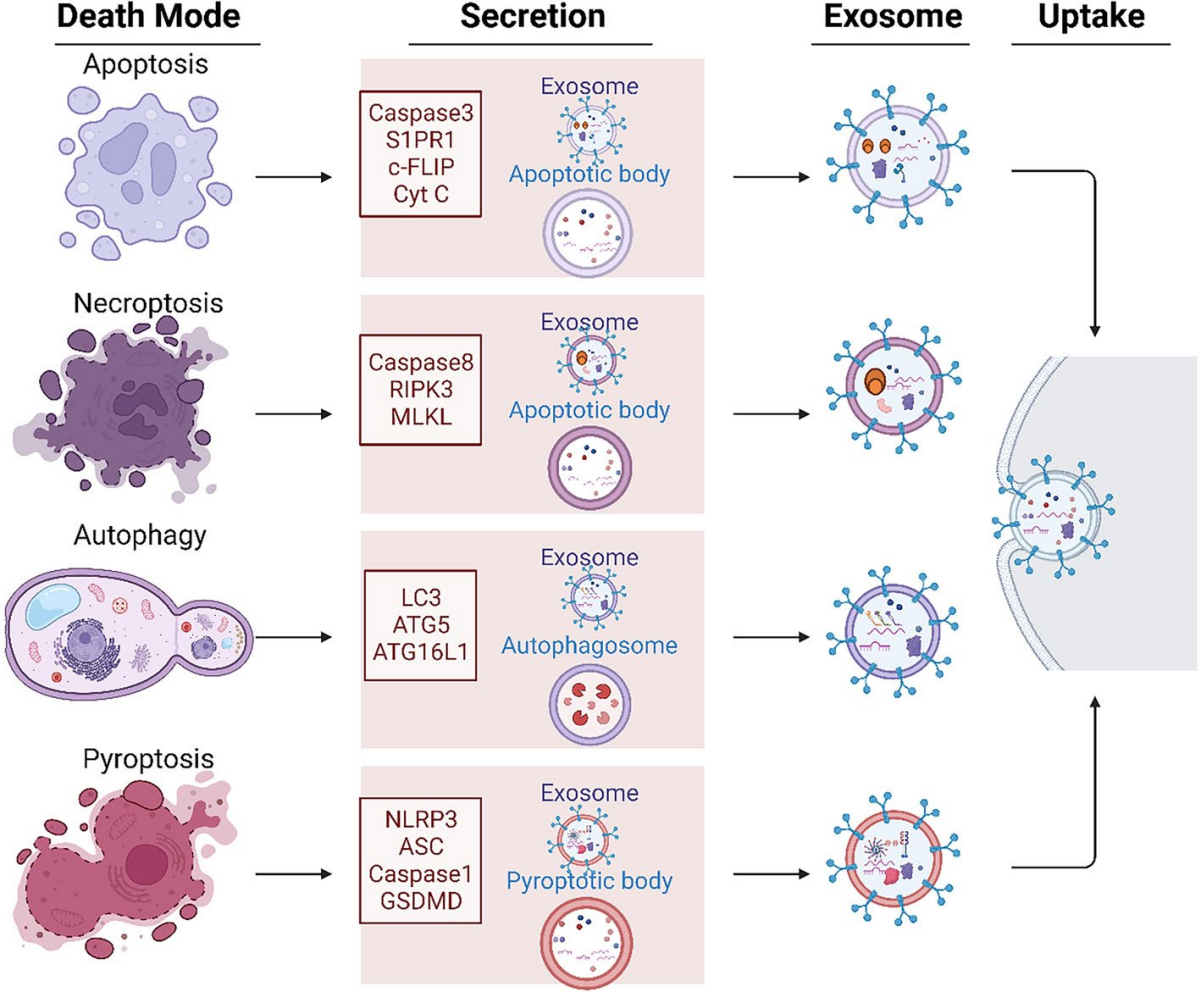
Exosomes derived from distinct programmed cell death pathways exhibit varied molecular compositions and exert diverse functional impacts. Throughout cellular apoptosis, key initiators such as Caspase 3 and Cyt C actively participate in regulating exosome secretion. Specifically, apoptotic exosomes (ApoExos) carry a distinctive protein set associated with apoptosis, including Caspase 3, S1PR1, c-FLIP, and Cyt C. This protein combination results in the formation of a unique secretory payload systematically released into the extracellular milieu. In contrast, necroptotic exosome secretion relies on specific proteins linked to necroptosis, including cysteine protease 8, RIPK3, and MLKL. Autophagic exosome secretion involves LC3 and ATG proteins, while pyroptotic exosome secretion involves NLRP3, ASC, and Caspase 1. Crucial components such as Caspase 3, Caspase 8, LC3, and NLRP3 play pivotal roles in regulating cell death and influencing exosome generation and bioactive molecule selection. This secretion mechanism not only displays apparent heterogeneity but also mirrors diverse molecular regulatory mechanisms involved in various cell death pathways, ultimately enhancing intercellular communication during cell death. In addition to exosomes, various secretory vesicles, including apoptotic bodies from apoptotic cells, autophagosomes from autophagic cells, and pyroptotic bodies from pyroptotic cells, are released through diverse programmed cell death mechanisms. However, these vesicles exhibit notable differences in size, composition, and functionality compared to exosomes. This diversity enriches cell death phenotypic features, providing a more intricate regulatory mechanism for intercellular information transmission

In summary, exosomes that cause programmed cell death exhibit diverse release mechanisms, compositions, and functions. They are produced by specific enzymes or pathways, encapsulating mediators for extracellular transport (Fig. 2). These exosomes modulate recipient cell functions, such as growth, differentiation, apoptosis, and immune activation, through endocytosis, membrane fusion, or receptor interaction [71]. This signaling pathway impacts the immune microenvironment and inflammation, contributing to diseases. For instance, exosomes from apoptotic neurons containing misfolded mutant SOD1 can trigger neuronal death and contribute to amyotrophic lateral sclerosis (ALS) [72]. Intestinal epithelial cells damaged by ischemia/reperfusion release exosomes that promote cortical neuron death [73]. Understanding the various programmed cell death modes and their molecular mechanisms is crucial for gaining insights into diseases and identifying therapeutic targets.

### Effects of programmed cell death on exosome release

#### Apoptosis and exosomes

Despite previous assumptions that apoptotic cells are “immunologically silent” due to nuclear shrinkage, cell blebbing, and suppressed immune responses, recent studies have revealed their ability to communicate with healthy cells through exosomes [40]. Apoptosis results in the destruction and degradation of cellular organelles and biomolecules. The resultant debris is encapsulated by the cell membrane, where it forms apoptotic bodies that contribute to the propagation of apoptotic signals and the stimulation of phagocytosis.

Apoptotic bodies and exosomes differ in size (typically 1000–5000 nm versus 30–150 nm), biogenesis, and composition. Research by Uyen et al. on extracellular vesicles, encompassing apoptotic bodies, microvesicles, and exosomes from HaCaT and PKC cells, revealed distinct miRNA expression patterns. Notably, hsa-miR-3614-5p was enriched in exosomes, while hsa-miR-29b-1-3p was more abundant in apoptotic bodies and microvesicles [74]. Exosomes, referred to as apoptotic extracellular vesicle-like exosomes (ApoExos), are released during apoptosis and can induce inflammation at vascular lesion sites. ApoExos include distinctive components, including C-terminal perlecan LG3 fragments and 20 S proteasome subunits [45], along with specific proteins such as sphingosine-1-phosphate receptors 1 and 3 (S1PR1 and 3). These exosomes can modulate apoptosis by transferring specific proteins and RNAs, either by inhibiting or promoting the process. For instance, Sirois demonstrated that ApoExos carry antiapoptotic proteins such as translationally controlled tumor protein (TCTP), leading to the inhibition of apoptosis. Caspase-3 activation regulates TCTP secretion, thereby stimulating exosome release to achieve antiapoptotic effects [75]. Conversely, Yu et al. discovered that exosomes from apoptotic MCF-7 breast cancer cells contain IL-6, which has proapoptotic effects and upregulates MMPs in naïve MCF-7 cells via the IL-6/STAT3 pathway, ultimately promoting proliferation and metastasis [76]. Additionally, Yao et al. noted heightened extracellular vesicle secretion in cells undergoing ER stress for immune evasion [77], while Gurunathan reported amplified exosome synthesis and release under conditions of oxidative stress, ER stress, and apoptosis induced by PdNPs [78].

**Fig. 3:**
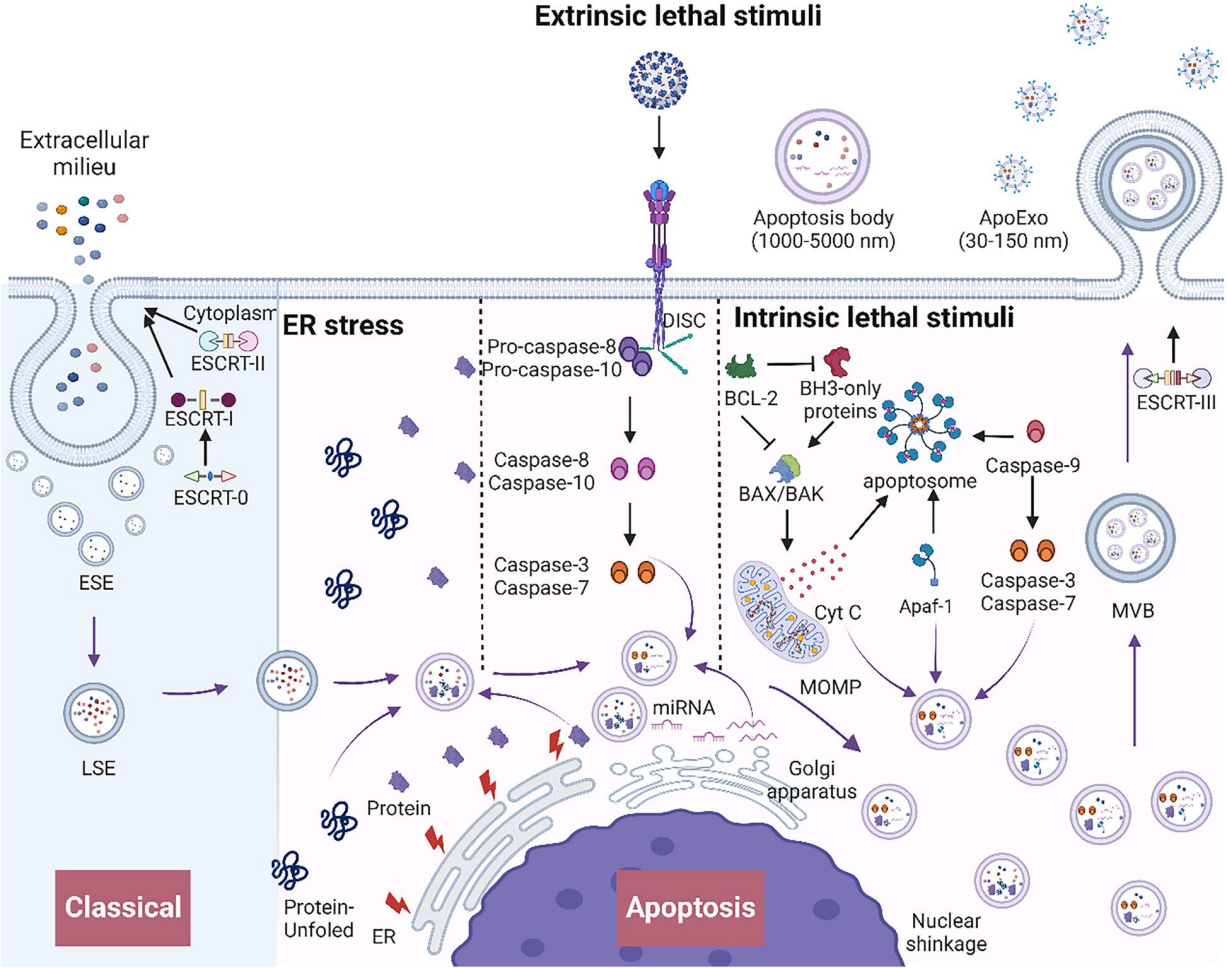
The biogenesis and release mechanisms of exosomes during apoptosis. Classical: The components of the endosomal sorting complex required for transport (ESCRT) actively participate in the formation of exosomes, a pivotal process in intracellular transport. The ESCRT-0 complex, characterized by the abundant HRS-STAM heterotetramer, initiates intracellular sorting and recruits ESCRT-I. Collaboratively, ESCRT-I and ESCRT-II orchestrate membrane invagination, giving rise to early endosomes (ESEs). These ESEs undergo mutual fusion, maturing into late endosomes (LSEs). Apoptosis: In ER stress-induced apoptosis, LSEs can recognize and encapsulate misfolded proteins within the endoplasmic reticulum. In exogenous apoptosis, membrane receptors such as tumor necrosis factor receptor 1 (TNFR1), death receptors, or Toll-like receptors (TLRs) are activated, resulting in the recruitment of the TNFR1-associated death domain (TRADD) or Fas-associated death domain (FADD), which interact with procaspase 8/10 to form the death-inducing signaling complex (DISC). Cleavage of procaspase 8/10 activates caspase 3/7, promoting LSE formation and cargo sorting, which are encapsulated within the endosomal lumen. In endogenous apoptosis, BAK and BAX activate proteins to form pores in the mitochondrial outer membrane, releasing Cyt C into the cytosol. Free Cyt C binds Apaf-1, activating pro-caspase 9 and forming the apoptosome complex. Caspase-9 is activated within the apoptosome to further activate caspase 3/7. Cyt C, Apaf-1, and activated caspase 3/7 can all facilitate late endosome formation and sequestration into the endosomal lumen. Ultimately, LSEs fuse into MVBs. With the assistance of the ESCRT-III complex, these MVBs release exosomes into the extracellular matrix by fusing with the plasma membrane. Additionally, apoptotic bodies are released during apoptosis. (Black arrows: involved in apoptotic mechanisms; purple arrows: involved in exosome biogenesis; curved purple arrows: involved in the formation of exosome cargo)

In summary, the release of ApoExos enables apoptotic cells to communicate with neighboring cells, thereby mediating the cellular microenvironment and influencing cell proliferation. However, the specific mechanisms regulating ApoExo release through the endosomal pathway during apoptosis have not been elucidated (Fig. 3).

#### Necroptosis and exosomes

**Fig. 4:**
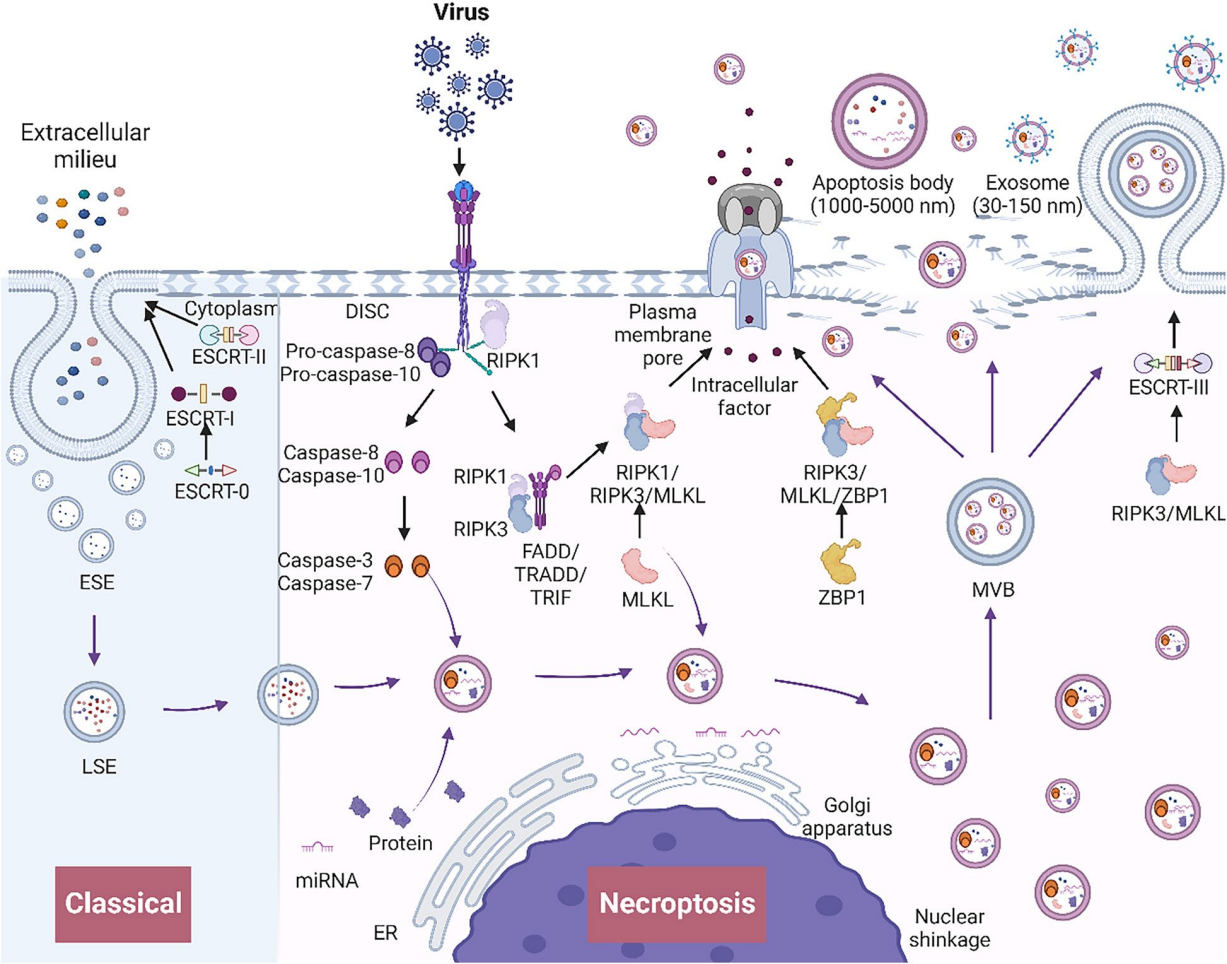
The biogenesis and release mechanisms of exosomes during necroptosis. Classical: The ESCRT-0 complex initiates intracellular sorting and recruits ESCRT-I. Collaboratively, ESCRT-I and ESCRT-II orchestrate membrane invagination, giving rise to early endosomes (ESEs). These ESEs undergo mutual fusion, maturing into late endosomes (LSEs). Necroptosis: In the initial stages of necroptosis, membrane receptors, such as tumor necrosis factor receptor 1 (TNFR1), death receptors, and Toll-like receptors (TLRs), are activated. This activation leads to the assembly of TNFR1-associated death domain protein (TRADD), Fas-associated death domain protein (FADD), receptor-interacting serine/threonine-protein kinase 1 (RIPK1), and pro-caspase 8/10, culminating in the formation of the death-inducing signaling complex (DISC). Subsequently, procaspase 8/10 undergo cleavage to yield mature caspase 8/10, thereby activating caspase 3/7. Activated caspase 3/7, in turn, promotes LSE formation and cargo sorting and becomes encapsulated within LSEs. In the late stage of necroptosis, a complex is formed in which RIPK1/RIPK3 phosphorylates mixed lineage kinase domain-like protein (MLKL), and ZBP1 directly binds RIPK3 and MLKL through its RHIM domain. MLKL proceeds to generate pore complexes in the membrane, causing membrane disruption. Concurrently, MLKL aids in the formation and cargo sorting of LSEs, which encapsulate itself within the endosomal lumen. Eventually, LSEs undergo fusion, evolving into multivesicular bodies (MVBs). Through RIPK3/MLKL-mediated ESCRT-III phosphorylation and membrane invagination, MVBs give rise to exosomes. Alternatively, exosomes may be passively released due to rupture of the cell membrane or actively expelled through MLKL pores in the membrane. In addition to exosomes, apoptotic bodies are also released during necroptosis. (Black arrows: involved in necroptosis mechanisms; purple arrows: involved in exosome biogenesis; curved purple arrows: involved in the formation of exosome cargo)

In necroptosis, the rupture of the cell membrane releases intracellular contents, thereby activating the immune system and inducing inflammation [6]. Necroptosis is characterized by an initial apoptotic phase followed by subsequent necrotic events. Apoptotic bodies are liberated during the apoptotic phase. As necrosis progresses, cells undergo biochemical self-dissolution, which promotes the release of exosomes under intracellular or extracellular pressure [3]. The exosomal contents are encapsulated by the cell membrane for discharge upon necrotic cell death. Yoon et al. [58] reported that mixed lineage kinase domain-like protein (MLKL) mediates endosomal protein transport via phosphorylation, thus facilitating exosome generation during necroptosis. Additionally, proteomic analysis by Shlomovitz et al. revealed that exosomes derived from necroptotic cells are enriched in inflammatory signaling components and tumor antigens and concurrently upregulate MLKL and caspase-8 [79]. Research on polychlorinated biphenyl (PCB) toxicity has demonstrated that PCB-induced necroptosis in cancer cells enhances exosome synthesis and release through MLKL phosphorylation MLKL. Encapsulated in exosomes, MLKL is extracellularly delivered to mitigate PCB-induced necroptosis [80]. Consequently, MLKL plays dual roles in necroptosis by regulating exosome secretion and providing feedback to modulate the necroptotic process (Fig. 4).

#### Autophagy and exosomes

Autophagy facilitates the long-distance transfer and recycling of proteolytic degradation products through exosome release [81]. In this intricate process, cellular proteins and metabolic waste are encapsulated within autophagosomes, which subsequently merge with lysosomes for digestion and discharge. Autophagosomes play a crucial role in maintaining cellular homeostasis, eliminating intracellular waste, and regulating cell growth.

**Fig. 5:**
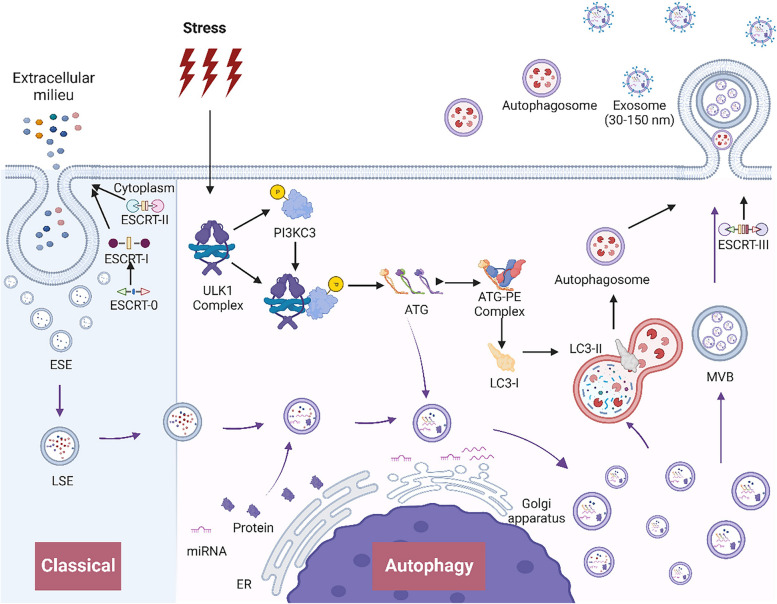
The biogenesis and release mechanisms of exosomes during autophagy. Classical: The ESCRT-0 complex initiates intracellular sorting and recruits ESCRT-I. Collaboratively, ESCRT-I and ESCRT-II orchestrate membrane invagination, giving rise to early endosomes (ESEs). These ESEs undergo mutual fusion, maturing into late endosomes (LSEs). Autophagy: During autophagy, the activation of ULK1, FIP200, and various ATG proteins leads to the formation of the ULK1 complex. This complex phosphorylates and binds to the PI3KC3 complex, initiating the activation of phosphatidylinositol-3-phosphate (PI3P) and recruiting ATG family proteins. Consequently, ATG proteins not only bind to membrane-resident phosphatidylethanolamine (PE) to catalyze the phospholipidation of LC3-I but also facilitate the formation of late endosomal structures (LSEs) and cargo sorting. These structures are eventually encapsulated by late endosomes. Ultimately, LSEs fuse into multivesicular bodies (MVBs), and MVBs undergo budding to release exosomes through ATG-mediated ESCRT-III phosphorylation and membrane invagination. In addition to exosomes, autophagosomes are also released during autophagy. (Black arrows: involved in autophagy mechanisms; purple arrows: involved in exosome biogenesis; curved purple arrows: involved in the formation of exosome cargo)

While both exosomes and autophagosomes are vesicular structures involved in molecular transport and degradation, they differ in terms of biogenesis and function. Exosomes are formed through membrane invagination and are released for intercellular communication. During their formation, exosomes may undergo fusion with lysosomes for degradation, lysosomal exocytosis for discharge, or release through inward budding [82]. Lysosomal dysfunction is known to augment exosome secretion [83], as does compromised autophagy [22]. In autophagy, lysosomal fusion with the plasma membrane facilitates lysosomal exocytosis and exosome release, a process regulated by caspase-3 [84]. Minakaki et al. observed the release of extracellular vesicles exhibiting an “autophagosome-exosome” phenotype during autophagy [85]. The colocalization of the autophagy marker LC3 with the exosome marker CD63 underscores the synergistic effects of autophagy and exosomes, enhancing macrophage recruitment and M1 polarization during viral infection [86]. Additionally, autophagy proteins such as ATG5 and ATG16L1 modulate exosome formation by regulating the intracellular pH and enriching exosomes with LC3-ATG5-ATG16L1 complexes [63]. The ATG12-ATG3 complex also contributes to secretion by interacting with the ESCRT protein Pdcd6ip (Alix) [63, 87, 88]. Thus, autophagy intricately influences exosome biogenesis, transport, and release through multiple mechanisms (Fig. 5).

#### Pyroptosis and exosomes

**Fig. 6:**
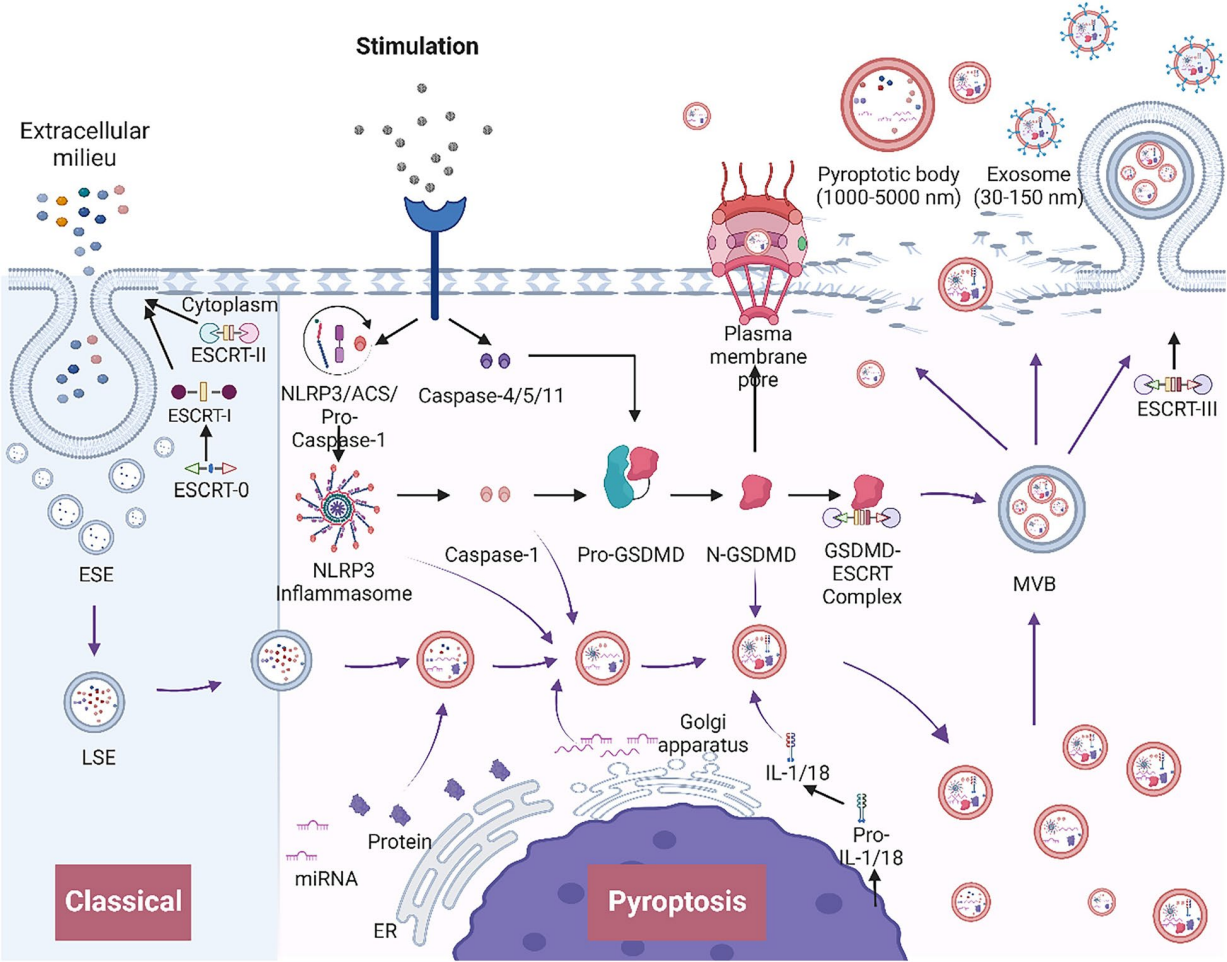
The biogenesis and release mechanisms of exosomes during pyroptosis. Classical: The ESCRT-0 complex initiates intracellular sorting and recruits ESCRT-I. Collaboratively, ESCRT-I and ESCRT-II orchestrate membrane invagination, giving rise to early endosomes (ESEs). These ESEs undergo mutual fusion, maturing into late endosomes (LSEs). Pyroptosis: NLRP3 oligomerizes and recruits pro-caspase 1 and ASC to assemble the NLRP3 inflammasome. Upon activation, caspase-1 in the NLRP3 inflammasome cleaves the substrate protein GSDMD. This cleavage results in the translocation of the N-terminal fragment of GSDMD to the plasma membrane, where it interacts with phospholipids and forms 10–15 nm pores. GSDMD can also form complexes with ESCRT. Cascanically, caspase-4/5/11 are directly activated to cleave GSDMD, resulting in the formation of pores in the membrane. The NLRP3 inflammasome, caspase-1, and N-terminal fragment of GSDMD can promote LSE formation and cargo sorting, leading to encapsulation in the endolysosomal lumen. Inflammatory cytokines such as IL-1β and IL-18 can also be encapsulated in LSEs. Ultimately, LSEs fuse into MVBs. MVBs release exosomes through GSDMD-ESCRT-driven membrane invagination. Exosomes can also be released by the ruptured cell membrane or actively released through GSDMD pores in the membrane. In addition to exosomes, pyroptotic cells also release pyroptosis bodies. (Black arrows: involved in pyroptosis mechanisms; purple arrows: involved in exosome biogenesis; curved purple arrows: involved in the formation of exosome cargo)

Pyroptosis, an inflammatory programmed cell death process, involves the release of exosomes containing inflammatory factors that impact the inflammation and immune responses of neighboring cells. Bulek et al. reported that gasdermin D (GSDMD), a crucial pyroptosis effector, collaborates with NEDD4 to release IL-1β-enriched exosomes, thereby triggering inflammation in patients with inflammatory bowel disease [89]. GSDMD facilitates pore formation on the cell membrane and regulates vesicle secretion. The pyroptosis regulator IQGAP1 associates GSDMD with ESCRT complexes during vesicle release, enabling pro-IL-1β vesicle secretion [90]. Moreover, cell membrane dissolution during pyroptosis also promotes exosome release [3] (Fig. 6).

Chen et al. demonstrated that zinc oxide nanoparticles and UV costimulation induce NLRP3 inflammasome-dependent pyroptosis and autophagy impairment in keratinocytes. Exosomes released by pyroptotic cells containing the NLRP3 inflammasome further stimulate inflammation in recipient keratinocytes [22]. These exosomes derived from pyroptotic cells are rich in IL-1β, IL-18, and other immune-modulatory factors [90–92]. However, not all pyroptotic exosomes exacerbate inflammation. In some contexts, regenerative neurons recruit and induce monocyte pyroptosis, leading to the release of osteopontin (OPN)-bearing vesicles from pyroptotic cells, which confer cytoprotection to neighboring cells [93].

### Biological functions of exosomes released during programmed cell death

#### Regulation of inflammation and the tumor microenvironment

Recent studies have emphasized the pivotal role of heterogeneous exosomes released by dying cells in intercellular communication, which is crucial for regulating inflammatory responses and remodeling the tumor microenvironment. These exosomes, enriched with bioactive molecules, facilitate diverse signaling pathways involved in inflammation. The regulatory impact of these proteins on inflammation is influenced by the origin of the secretory cell, its status, and the exosomal content. For instance, exosomes from inflammatory cells can enhance the release of inflammatory mediators and activate relevant pathways, thereby modulating the inflammatory milieu. In addition, active inflammatory responses can also modify the biogenesis, release, and physicochemical properties of exosomes. A study by Asheed et al. revealed that asthmatic reactions can not only trigger cascades of cytokine release but also promote exosome biogenesis and secretion. Additionally, compared with those in the normal control group, the exosomes isolated from the bronchoalveolar lavage fluid (BALF) of asthma patients were larger [94].

Specifically, exosomes from cells undergoing distinct programmed cell death modes exhibit unique characteristics and functions. Apoptotic cells release exosomes that convey proapoptotic signals to adjacent cells or that carry anti-inflammatory molecules such as IL-10 and TGF-β, in addition to immunosuppressive proteins. These components work to dampen inflammation, regulate immune cell activation, and prevent excessive inflammatory responses. Additionally, these exosomes may contain molecules that negatively regulate inflammatory signaling, contributing to immune homeostasis.

Conversely, exosomes from cells undergoing pyroptosis or necroptosis typically enhance inflammatory signaling, potentially leading to a cytokine storm and exacerbating inflammation [22]. Understanding the differential composition and release mechanisms of exosomes in various programmed cell death pathways is crucial for advancing our knowledge of inflammatory processes and identifying new therapeutic avenues for treating inflammatory conditions.

In addition to their role in inflammation, exosomes from cells undergoing different programmed death modes also influence the tumor microenvironment. They carry biomolecules that affect tumor cell behaviors, including proliferation, invasion, and metastasis, thus impacting tumor progression and response to treatment. Exosome-based cancer immunotherapeutic strategies involve blocking exosome generation from tumor cells, leveraging native exosomes, and engineering exosomes for intervention. However, the heterogeneity and diverse physicochemical properties of exosomes remain major challenges for their clinical translation [17]. Deciphering the molecular composition and actions of these exosomal subtypes is essential for understanding the regulatory dynamics of the tumor microenvironment and developing targeted therapeutic strategies.

However, the specific mechanisms by which key proteins in cell death pathways, such as caspase-3, RIPK3, ATG, and GSDMD, modulate immunity, inflammation, and the tumor microenvironment have not been fully elucidated.

#### Clearance of metabolites

Cell death plays a pivotal role in maintaining tissue homeostasis and normal physiological functions. In programmed cell death, cellular organelles undergo gradual degradation, ultimately leading to cell disintegration. The resulting cellular components can either passively leak into the extracellular environment due to membrane disruption or be encapsulated into exosomes for organized release. While lysosomes digest certain cell fragments during autophagy, other fragments are enveloped in vesicles and released through lysosomal exocytosis or membrane fusion [83]. This vesicular release is critical for efficiently clearing cellular debris, thereby preventing inflammation or necrosis [5]. Additionally, the uptake of these vesicles by recipient cells facilitates metabolite recycling and triggers immune responses, which are essential for pathogen elimination, inflammatory regulation, and sustained immune homeostasis.

#### Tissue repair and regeneration

Exosomes are released during cell death, carry specific signaling molecules and nutrients, and induce a reparative state in surrounding cells, promoting cell proliferation, differentiation, and migration. These exosomes contain proteins that synthesize molecules such as collagen, elastin, matrix metalloproteinases, and various extracellular matrix components, all of which are crucial for tissue repair [40]. Furthermore, exosomes serve as communication vehicles, delivering growth factors, cytokines, and regulatory molecules to recruit immune cells, such as dendritic cells, macrophages, natural killer cells, myeloid-derived suppressor cells, and T cells, to damaged sites [95]. These immune cells play key roles in removing dead cells and debris and secreting various factors, including platelet-derived growth factors, TGF-β, interferons, and interleukins, which orchestrate the proliferation, differentiation, and migration of adjacent cells, contributing to effective tissue repair. Thus, exosome-mediated intercellular communication is integral to tissue repair processes during programmed cell death.

### Application

#### Disease biomarkers and therapeutic targets

Exosomes hold promise in the diagnosis and treatment of human diseases. Isolating exosomes from diverse body fluids, such as blood [96], urine [97], and breast milk [98], facilitates the detection of disease-associated biomolecules, including proteins and nucleic acids. Notably, Shadi et al. demonstrated that exosomes derived from senescent cells contain biologically active molecules implicated in cell proliferation, including P53, P21, P16, FMR1, and miR-21. These exosomes may impact the proliferation and migration of target cells in coculture. Thus, exosomes derived from senescent cells may serve as potential biomarkers for age-associated vascular diseases [99]. Profiling specific biomarkers enables the use of exosomes in diagnostic, therapeutic monitoring, and disease screening [100]. Exosomal analysis, which has advantages over traditional diagnostic methods, is noninvasive and convenient and yields stable results, facilitating early disease detection and improving treatment outcomes.

As byproducts of programmed cell death, exosomes also function as indicators of disease progression, assisting in diagnosis and evaluating treatment response by identifying unique molecular markers. Nevertheless, further research is needed to comprehensively comprehend the roles of exosomal biomarkers and their potential clinical applications. In summary, exosomes produced during programmed cell death are instrumental in disease pathogenesis. Formulating strategies targeting these extracellular vesicles may present novel avenues for disease prevention and therapy [101]. Characterizing disease-specific exosome subpopulations and elucidating their molecular content can lead to the validation of these vesicles as biomarkers in patient biofluid samples, creating new possibilities for diagnosis and treatment.

#### Targeted therapy

Exosomes, which serve as inherent nanocarriers, possess a lipid bilayer membrane that potently shields their internal contents from degradation. Additionally, their restricted surface proteins diminish immunogenicity and lessen the probability of immune recognition and phagocytosis. Remarkably, exosomes can traverse the blood‒brain barrier [102], establishing them as versatile drug delivery vehicles for treating various diseases, such as cancer, cardiovascular disease, and neurodegenerative disorders.

Earlier investigations have leveraged exosomes derived from mesenchymal stem cells for immunotherapy, predominantly by modulating immune responses, augmenting cell proliferation and repair, and mitigating inflammation. Recent investigations, however, have revealed the potential of employing exosomes released during programmed cell death for targeted drug delivery, suggesting a novel therapeutic strategy. For instance, determining the composition of exosomes in autophagic cells can augment bioavailability, efficacy, and specificity [81]. Exosomes can be precisely targeted through genetic engineering or chemical modification to introduce specific proteins or microRNAs or to alter their membrane composition and structure [103]. An illustrative example is the study by Zhou et al., in which tumor cell-derived exosomes were extracted and loaded with CCL22 siRNA (spMEXO), leading to the successful inhibition of DC/Treg interactions and Treg expression, subsequently impeding tumor growth and providing novel possibilities for cancer treatment [104].

### Challenges and future prospectives

Exosomes, renowned for their unique properties, such as nontoxicity, low immunogenicity, hydrophilic core, high delivery capability, and specific targeting, prove valuable as nanocarriers in targeted drug delivery applications. Nevertheless, the practical utility of exosomes is constrained by their limited secretion capacity. Despite the complexity of their application, exosomes derived from cells undergoing programmed cell death lack specific isolation techniques and identifiable markers, posing challenges in effective purification and identification. The exosomes extracted from these cells may originate from various sources, including normal cells, cells that initiate death, or cells that complete the death process, resulting in a heterogeneous mixture that is challenging to separate and purify.

Engineered exosomes generated during the programmed cell death process are increasingly emerging as promising tools in targeted drug delivery and precision nanomedicine. Future research should primarily focus on elucidating the biological characteristics of exosomes, accurately identifying their specific markers, and designing specialized methods for isolation and identification to lay the groundwork for industrial-scale preparation and clinical application. | Subsequent research efforts should concentrate on optimizing exosome isolation, cargo loading, and surface modification techniques to comprehensively exploit their potential therapeutic applications. These advancements are crucial for fully realizing the potential of exosomes in medical applications.

## Conclusion

Exosomes are ubiquitously released by most cell types; however, their specific composition and functional effects are modulated by the activation state of parent cells and microenvironmental stimuli. When cells undergo programmed cell death, key regulatory proteins also control exosome generation and release, which encapsulate cell death signals within exosomes for intercellular communication. Exosomes originating from distinct programmed cell death pathways exhibit marked heterogeneity and can dynamically regulate physiological and pathological processes. For example, while apoptotic cells were previously considered immunologically silent, they can signal intercellularly by releasing exosomes with a distinct molecular repertoire that includes Caspase 3, S1PR1, c-FLIP and Cytochrome C to influence inflammation and apoptosis in recipient cells. In necroptosis, cell membrane rupture promotes the release of proinflammatory exosomes generated primarily through an MLKL-dependent pathway that encapsulate cysteine protease 8, RIPK3 and MLKL. Impaired autophagy can escalate exosome release via lysosomal exocytosis, enriching exosomes with LC3 and ATG proteins. Moreover, pyroptotic cells discharge exosomes via a GSDMD-dependent mechanism involving NLRP3, ASC and Caspase 1 to propagate inflammatory signals. The exosomes released during programmed cell death can regulate inflammation, influence the tumor microenvironment, clear metabolites, and promote tissue repair and regeneration. Specific exosomal subpopulations may also serve as disease biomarkers, providing diagnostic and therapeutic possibilities. Although the isolation and identification of exosomes from distinct cell death pathways pose challenges due to marked heterogeneity, gaining deeper insight into their composition and functions is critical for elucidating their roles in inflammation, immune regulation, tissue repair, and intercellular signaling. Comprehensive profiling of the biological traits of exosomes originating from programmed cell death modalities would enable clinical applications involving their use as biomarkers and for targeted drug delivery. In summary, as dynamic communicators in programmed cell death, exosomes warrant extensive investigation to fully harness their significant roles in biological processes.

## Data Availability

No datasets were generated or analysed during the current study.
